# Comparative pathogenesis, tissue tropism, and immune dynamics of infectious bronchitis virus genotypes GI‑1 and GI‑24 in broiler chickens

**DOI:** 10.1016/j.psj.2026.107782

**Published:** 2026-09-15

**Authors:** Most. Shahana Akter, Mohosin Kabir, Md. Riabbel Hossain, Shadia Tasnim, Farhana Firoze Promi, Rupaida Akter Shila, Jahan Ara Begum, Emdadul Haque Chowdhury, Rokshana Parvin

**Affiliations:** Department of Pathology, Faculty of Veterinary Science, Bangladesh Agricultural University, Mymensingh, 2202, Bangladesh

**Keywords:** Infectious bronchitis virus, Genotype GI 1 and GI 24, Pathogenesis, Cytokine dynamics, Immune response

## Abstract

Infectious bronchitis (IB) is an acute and highly contagious respiratory and renal disease of poultry caused by infectious bronchitis virus (IBV). This study comparatively evaluated the pathogenesis, tissue tropism, viral shedding, cytokine dynamics, and humoral immune responses induced by the recently characterized GI-1 and GI-24 variant strains of IBV in broiler chickens. Birds (*n* = 90) were experimentally inoculated intranasally and intraocularly with 10⁶ EID₅₀/0.1 ml of either GI-1 (*n* = 30) or GI-24 (*n* = 30), while control birds (*n* = 30) received uninoculated allantoic fluid and were monitored for 21 days post-infection (dpi). Both IBV variants induced pronounced clinical disease characterized by pyrexia, reduced weight gain, severe respiratory and renal manifestations. Gross and histopathological examinations revealed marked congestion, hemorrhage, tubular degeneration, and necrosis in lungs and kidneys of infected birds. Real-time RT-PCR analysis demonstrated high viral loads and prolonged viral shedding in both respiratory and renal tissues, persisting through 21 dpi (*P* < 0.001) accompanied by distinct tissue-specific inflammatory and pro-inflammatory cytokine responses representing the first characterization of organ-specific and swab-associated cytokine dynamics following GI-24 infection. The GI‑1 variant consistently induced higher and more sustained expression of pro‑inflammatory cytokines; IL‑1β and TNF‑α (*P* < 0.001 and *P* < 0.01), antiviral interferons; IFN‑γ and IFN‑β (*P* < 0.01), and the regulatory cytokine IL‑10 (*P* < 0.01) compared with GI‑24. Both infected groups developed robust humoral immune responses with seroconversion by 14 dpi and peak antibody titers at 21 dpi. Overall, GI-1 exhibited greater pathogenicity and more persistent lesions than GI-24, whereas the emerging GI-24 variant showed dual respiratory and renal tropism and induced distinct tissue-specific cytokine responses, representing the first detailed characterization of its immunopathogenesis and highlighting the need for continuous surveillance and strain-matched vaccination strategies.

## Introduction

Infectious bronchitis (IB) is a highly contagious viral disease of poultry that poses a great threat to the poultry industry worldwide, causing substantial economic loss due to decreased productivity, poor weight gain, and mortality (Uddin et al., 2025). It is caused by the Infectious bronchitis virus (IBV), which is designated as Avian coronavirus (AvCoVs) under the family Coronaviridae and the genus Gammacoronavirus, characterized by a single-stranded, positive-sense RNA genome approximately 27.6 kb in length (King et al., 2018). The viral genome encodes many structural proteins, including the spike (S), envelope (E), membrane (M), and nucleocapsid (N) proteins (Chen et al., 2021; Parvin et al., 2021). The spike protein, especially the S1 subunit, is the major protein for viral attachment, neutralizing antibodies against the host immune response and is responsible for antigenic diversity of IBV (Valastro et al., 2016). The disease was first identified in the United States in the 1930s and, since then, has been reported in all major poultry-producing regions, making it one of the most significant viral diseases in chickens (Brown et al., 2016; Promkuntod, 2016). Due to the error-prone nature and frequent recombination events, IBV exhibits high genetic variability, resulting in the emergence of many distinct genotypes and variants with variable pathogenicity and tissue tropism (Jackwood, 2012; Abdel-Moneim et al., 2012; Promkuntod et al., 2014; Kabir et al., 2026).

Currently, there are seven genotypes (GⅠ-GⅦ) comprising 35 variants (Valastro et al., 2016; Mase et al., 2022; Rafique et al., 2024). Among them, the GⅠ−1 genotype, also known as the Massachusetts type, is one of the most prevalent worldwide due to its use in live attenuated vaccines (Jackwood, 2012). This Mass type was previously associated with respiratory ailments; however, it alters tissue tropism, and pathogenicity may be due to the emergence of novel variants (Rafique et al., 2024; Saleem et al., 2024) .

Among the IBV genotypes, GI-24 has emerged as an important lineage in South and Southeast Asia. GI-24-related viruses have been reported in India and Pakistan, with recent phylogenetic evidence suggesting regional circulation and geographic expansion of this lineage (Rafique et al., 2024; Saleem et al., 2024). In Bangladesh, GI-24 was recently identified and clustered closely with Indian reference strains (Kabir et al., 2026), suggesting a possible regional introduction of this lineage. Although GI-24 viruses are associated with nephropathogenic disease, their tissue tropism and pathogenicity may vary among strains. Genetic recombination is an important mechanism driving IBV evolution and can alter viral tissue tropism and pathogenicity; however, whether recombination specifically accounts for the altered tissue tropism observed in GI-24 viruses remains unclear and requires comparative analysis.

Clinically, respiratory-associated IBV infection is characterized by respiratory distress, like coughing, sneezing, and nasal discharge, and tracheal rales (Rafique et al., 2024; Saleem et al., 2024). Some variants are nephropathogenic and cause interstitial nephritis, nephrosis resulting in pale, swollen kidneys, watery droppings, and urate deposition in the ureters (Nguyen et al., 2020). The diversity and severity of clinical signs are influenced by the variant of the virus, co-infection, environmental factors, and the flock's immune status (Ren et al., 2020; Tang et al., 2022).

In Bangladesh, infectious bronchitis continues to cause recurrent outbreaks in commercial broiler flocks despite the widespread use of classical vaccine strains. Previous molecular characterization of Bangladeshi IBV isolates revealed genetic and predicted antigenic differences between emerging GI-24 viruses and commonly used vaccine strains, particularly in S1 hypervariable and predicted neutralizing epitope regions (Kabir et al., 2026). These findings suggest potential antigenic divergence and reduced heterologous protection; however, vaccine efficacy cannot be confirmed from sequence differences alone (Bhuiyan et al., 2019; Uddin et al., 2025). Previously, Mass-like (GI-1), QX-like (GI-19), and 4/91-like (GI-13) variants of infectious bronchitis virus (IBV) were identified in Bangladesh (Parvin et al., 2020). Recent field investigations have identified the dominant circulation of GI-1 lineages alongside the emergence of the GI-24 genotype, a newly introduced variant in the country (Kabir et al., 2026), raising concerns regarding its pathogenic potential.

Although GI-1 variants are often considered vaccine-related or recombinant strains with limited pathogenicity, increasing evidence suggests altered biological behavior, including enhanced tissue tropism and disease severity. Conversely, GI-24 variants have been traditionally classified as nephrotropic; however, their pathogenic and immunological behavior under local production conditions has not been systematically evaluated. The lack of comparative experimental data on these variants under conditions representative of Bangladeshi poultry production systems constitutes a critical knowledge gap. Addressing this gap is essential for understanding variant-specific disease mechanisms, refining vaccination strategies, and mitigating production losses associated with IBV outbreaks.

This study was designed to compare the pathogenicity of two infectious bronchitis virus (IBV) variants belonging to the GI-1 lineage the predominant circulating variant and GI-24, a newly introduced variant, both isolated from clinical outbreaks in commercial broiler chickens in Bangladesh. Experimental infections were conducted in broiler chickens under controlled open-shed conditions representative of typical poultry production systems in Bangladesh. Therefore, the present study was undertaken to comparatively evaluate the pathogenicity, tissue tropism, and immune responses elicited by GI-1 and GI-24 IBV variants in experimentally infected broiler chickens, thereby providing evidence-based insights to support improved IBV control measures in Bangladesh. Additionally, this study contributes to the current understanding of IBV epidemiology, transmission dynamics, and pathogenic potential among domestic poultry populations in Bangladesh.

## Materials and methods

### *Viral strains*

The two recently characterized Infectious bronchitis virus (IBV) isolates of GI-1 (B53) and GI-24 (B60) lineage were received from the Department of Pathology, Bangladesh Agricultural University, Mymensingh 2202 (Bangladesh). Genotype Identification was performed (Kabir et al., 2026) by comparing amplified fragments of the spike 1 (S1) gene with the sequence available in the NCBI GenBank database under the accession numbers PX938991-PX939006. The genomic analysis demonstrated that strain B53 of genotype GI-1 shares 98.6–100% nucleotide identity with the commercially used vaccine strains Ma5, H120, and B-48, whereas strain B60 of genotype GI-24 is more genetically divergent, showing only 96.86% nucleotide identity with its closest reference strain and greater divergence from all vaccine strains. Furthermore, amino acid alignment revealed unique substitutions in the GI-24 isolate, including D38N and G64E within the S1 hypervariable regions, as well as sequence differences in the predicted neutralizing epitope region (residues 87–93) compared with both GI-1 and vaccine strains ((Kabir et al., 2026).

Both the GI-1 (B53) and GI-24 (B60) isolates were propagated in 9–10-day-old specific-pathogen-free embryonated chicken eggs, and allantoic fluid collected after the third consecutive embryo passage was used for virus titration and preparation of the challenge inoculum. The 50% embryo infectious dose (EID₅₀) was determined by serial 10-fold dilution. To address concerns regarding potential egg-adaptation attenuation, the S1 gene sequences of the passaged viruses were compared with those of the original field isolates and were found to be 100% identical, indicating that no detectable genetic changes occurred during egg propagation. Therefore, the pathogenicity data generated in this study are considered representative of the original field strains.

### *Experimental chicken*

A total of 90 commercial broiler (Cobb-500) chicks were raised from day-old in relative isolation with strict biosecurity measures. The food and water were supplied *ad libitum*. At 9 days of age, 10 serum samples were collected randomly from the birds and checked for the presence of maternal-derived antibodies against IBV using a commercial ELISA kit (ID Screen® Infectious Bronchitis Indirect, IDVet, Grabels, France). All the chicks were found seronegative against IBV.

### *Experimental design and infection*

The seronegative 9-day-old chickens of approximately the same weight and apparently healthy commercial broilers (*n* = 90) were randomly divided into 3 groups and kept in separate isolation pens. The group was named Control (*n* = 30), GI-1 IBV (*n* = 30), and GI-24 IBV (*n* = 30). At 10 days of age, the control group was infected with 100 μl sterile PBS through the intranasal and intraocular routes. Chicken from the GI-1 and GI-24 IBV groups were infected with 100 μl of titrated IBV (10^6^ EID_50_/0.1 ml) of GI-1 and GI-24 genotypes, respectively, through the intranasal and intraocular routes with an equal amount. All birds were closely observed for clinical signs and mortality twice daily up to 21 days post-infection (dpi). Oropharyngeal and cloacal swabs were taken at 3, 5, 7, 10, 14, 18, and 21 dpi. Blood was collected for serum analysis on dpi 7, 14, and 21. Tissue samples were collected at necropsy on dpi 3, 5, 7, 10, 14, 18, and 21 for histopathological and molecular detection studies (Fig. 1).

**Fig. 1:**
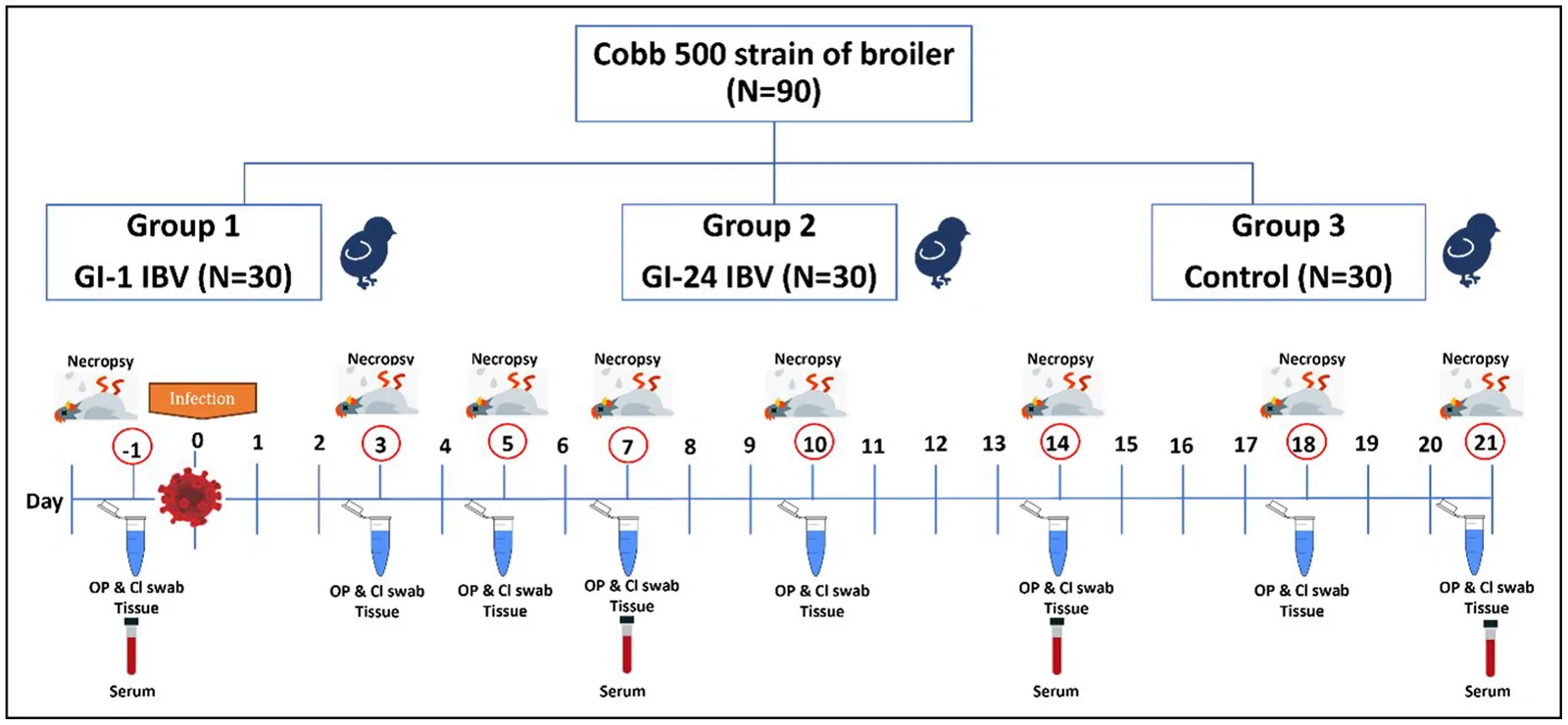
An experimental work design for a comparative pathogenicity study. Three different groups, each containing 30 birds, were designated as GI-1 (GI-1 IBV local variant treated), GI-24 (GI-24 IBV local variant treated), and control (negative allantoic fluid treated). Three replicates of birds from each group were selected for sample collection and deceased for necropsy at each mentioned (red round circle) time points.

### *Clinical examination*

All the chickens from both the infected and control groups were observed twice daily for clinical signs and mortality. Five birds from each group were randomly selected, and body weight and temperature were measured at 3, 5, 7, 10, 14, 18 and 21 dpi. For each group, daily clinical signs were reported separately.

### *Sampling and necropsy*

At 3, 5, 7, 10, 14, 18, and 21 dpi, oropharyngeal (OP) swabs were collected and pooled randomly from five birds for each group. Cloacal (CL) swabs were taken in the same way at the same dpi. Swab samples were preserved in antibiotic-added PBS and kept in −80 °C until use. Three birds from each group were randomly selected and euthanized for necropsy examination at every 3, 5, 7, 10, 14, 18 and 21 dpi. At necropsy, the gross pathological changes in different organs were recorded with special focus on the trachea, nasal turbinate, lung and kidney. Tissue samples from the nasal turbinate, trachea and lungs were collected and pooled as respiratory tissue and kidneys were taken in a sterile tube from each group at every dpi. Tissue samples were preserved at −80 °C for molecular studies. In addition, the lung and kidney were taken from each three groups at each dpi in 10% neutral buffered formalin for histopathological studies.

### *Histopathology*

10% neutral buffered formalin fixed lung and kidney tissues from each three groups were processed with standard histological techniques, embedded in paraffin, sectioned by microtome into 4 μm and mounted on a glass slide. The cut sections were then stained with haematoxylin and eosin (H & E) according to a previously described procedure (Akter et al., 2025). The stained tissue sections were visualised under a microscope (Olympus BX43, Centre Valley, PA, United States). The histopathological lesions were recorded.

### *Detection by RT-qPCR and viral load quantification*

Tissue samples (respiratory tissues and kidney) were disrupted using a Tissue Lyser (Qiagen, Hilden, Germany) and homogenised in lysis buffer at a 1:10 w/v ratio. Homogenates were centrifuged at 8000 rpm for 5 min, and the supernatant was used for viral RNA extraction to detect IBV. Viral ribonucleic acid (RNA) was extracted from the tissues and swab samples using GeneJET RNA Purification Kit (Thermo Fisher Scientific, USA) in accordance with the manufacturer’s instructions. Final elution was achieved in 100 μL of elution buffer. RNA concentration was measured with a Nanodrop one (ThermoScientific™, USA). RNA was stored at −80°C until further processing.

Single-step RT-qPCR assays were performed with previously designed unique oligonucleotide primers for the detection of the untranslated regions (5′-UTR) and a fragment of the polymerase gene of avian gammacoronaviruses (AvCoV) in a single assay (Parvin et al., 2021). Luna Universal One-Step RT-qPCR Kit (New England Biolabs Inc., USA) containing SYBR Green reagents was used following a previously standardized protocol (Uddin et al., 2025). The final reaction volume was 12.5 μl, including 2.5 RT-PCR SYBR reaction mix, 0.5 μ μl of the RNA template, 5 μl of 2 × l of Luna WarmStart RT Enzyme Mix, 2.5 μ l of nuclease-free water, and 2 μl of primer mix (10 pmol each). The RT-qPCR thermocycling conditions were 55°C for 30/45 min (reverse transcription) and 95 cycles at 95°C for 10 min (reverse C for 2 min (initial denaturation), followed by 45°C for 10 s (denaturation) and 60°C for 30 s (annealing and elongation) with a reading of fluorescence in this step. Immediately after PCR, a melting curve analysis was performed with a continuous temperature increment of 0.5°C/s between 65°C and 95°C. After completion of the assay, the amplification curves were viewed, and the cycle threshold (Ct) value and melting temperature (Tm) were recorded. A Ct value of < 35 was used as a threshold for positive results (Kabir et al., 2026).

The viral genome load was quantified using the standard curve generated from tenfold serial dilutions of a known concentration of infectious bronchitis virus (IBV) RNA (Callison et al., 2006). The standard curve was constructed by plotting the cycle threshold (Ct) values obtained from the real-time RT-qPCR assay against the corresponding log₁₀ RNA copy numbers. The calculated log₁₀ RNA copy numbers were used to express the viral genome load using the equation derived from the standard curve.

### *Quantitative analysis of cytokine responses following IBV infection*

Total RNA extracted from various tissue samples (Respiratory tissues and kidney) collected at different days post infection (dpi) was used for cDNA synthesis with the ABScript II cDNA First Strand Synthesis Kit (ABclonal Technology^TM^, USA) following the manufacturer’s instructions. After the measurement of the concentration of the synthesized cDNA using Nanodrop one (Thermo Scientific™, USA), it was diluted at a ratio of 1:10 using nuclease-free water. Afterward, the diluted cDNA was subjected to qPCR to assess the relative expression of interleukin-1β (IL-1β), IL-10, tumor necrosis factor-α (TNF-α), interferon- β (IFN-β) and IFN-ℽ in the respiratory tissues and kidneys collected from control and infected groups using published primers (Nang et al., 2011). The internal control during qPCR reactions was glyceraldehyde-3-phosphate dehydrogenase (GAPDH). The analysis was based on changes in SYBR Green fluorescence after amplification with specific primer sets (Supplementary Table 1) following the designated thermal profile: Initial Denaturation at 95 °C for 10 min followed by 45 cycles of denaturation at 95°C for 15 s, annealing at 50−53°C (based on cytokine genes used) for 1 min and extension at 72°C for 1 min. The 10 µl reaction was run in triplicates with Luna Universal qPCR Master Mix (5 µl) (New England Biolabs, Inc.), 5 pmol of each primer (1 µl) for each gene, and 100 ng of cDNA (2 µl) and nuclease-free water (1 µl). One cycle for the dissociation curve for all reactions was added, and the melting curve was analyzed. The data obtained from the relative qPCR of cytokine genes were subjected to the 2-ΔΔCT method (Livak and Schmittgen 2001), and the relative fold change in mRNA encoding respective cytokine genes between control and infected chickens was subsequently assessed (Hossain et al., 2025).

### *Monitoring of immune response after infection*

Blood was taken from five random birds from each Control, GI-1 IBV and GI-24 IBV groups and serum was collected to measure the immune response against IBV. A commercial enzyme-linked immunosorbent assay (ELISA) kit (ID Screen® Infectious Bronchitis Indirect, IDVet, Grabels, France) was used to measure the antibody titres against IBV according to the manufacturer’s recommendations. Titers greater than 1625 (cut-off value) were considered positive.

### *Statistical analysis*

All statistical analyses were performed using GraphPad^TM^ Prism version 10.6.1 (GraphPad Prism Software, San Diego, California, USA). Two-way analysis of variance (ANOVA), followed by Turkey’s multiple comparison test, was employed to analyse results. The results were expressed as the mean ±standard error of the mean (SEM). The difference between groups was considered significant at *P* < 0.05.

## Result

### *Clinical manifestations*

Throughout the 21-day observation period, both GI-1 and GI-24 IBV groups exhibited a significant rise in rectal temperature from 3 dpi onward presented in Fig. 2A. Temperature levels continued to rise for both groups up to 10 dpi, while the control group maintained a normal temperature (105 °F) throughout the study. Birds infected with the GI-1 showed slightly higher and persistent fever peaking at 107°F compared to GI-24 IBV, which peaked around 105°F. After 10 dpi, the temperature of both groups began to decrease and returned to normal levels by 21 dpi, indicating recovery.

**Fig. 2:**
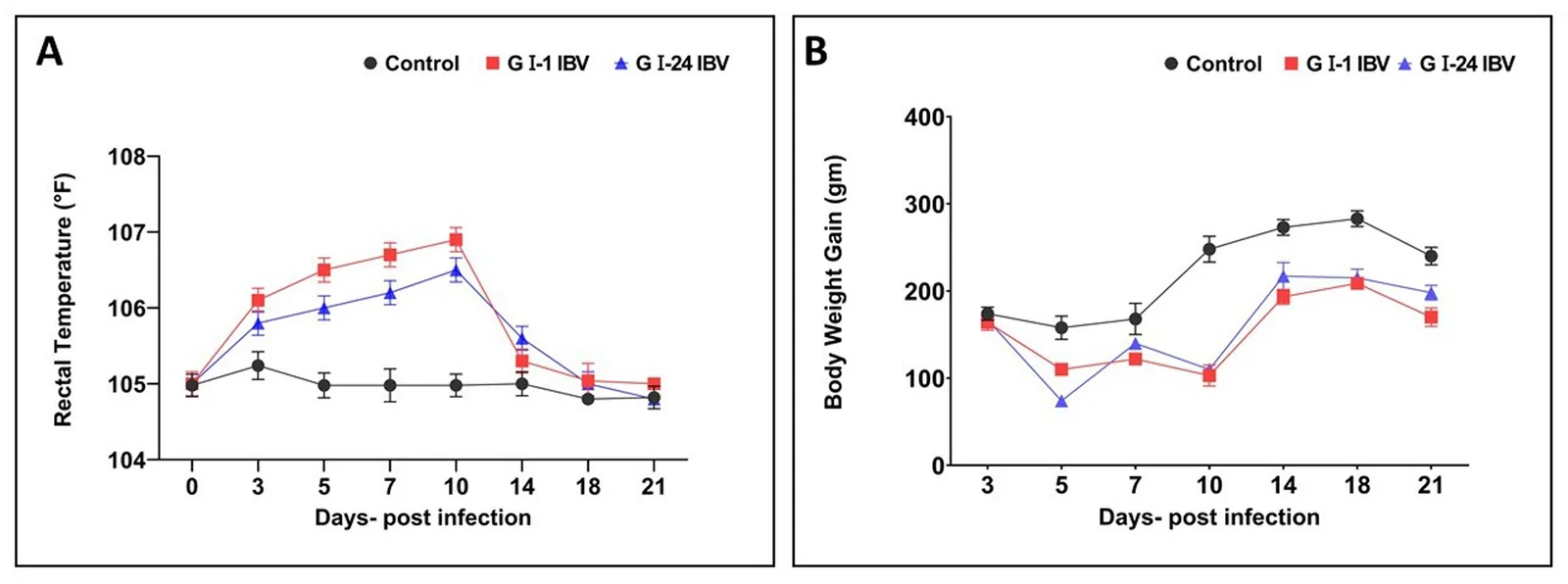
Rectal temperature **(A)** and body weight gain **(B)** profiles of chickens infected with the GI-1 and GI-24 variants of IBV compared with the control group. Data was presented as mean ± SEM.

The body weight gain of the GI-1 and GI-24 IBV groups and the control group from 3 dpi to 21 dpi is presented in Fig. 2B. A significant body weight gain was observed in both GI-1 and GI-24 IBV groups compared to the control group. At 3 dpi, similar body weight gain between the three groups indicates no initial difference before the onset of infection. However, by 5 dpi, the body weight gain of the GI-1 and GI-24 IBV groups declined remarkably compared to the control group. By 7 and 10 dpi, both infected groups continued to show reduced body weight gain, whereas the control group maintained a steady upward trend. After 10 dpi, a marked improvement in weight gain was observed for the rest of the time points. Overall, the control group maintained the highest weight gain, followed by the GI-24 and then the GI-1 IBV group.

The control group showed no clinical signs throughout the observation period. Chicken infected with GI-1 and GI-24 genotypes typically displayed a combination of respiratory and renal symptoms. These symptoms include sneezing, coughing, gasping, nasal discharge, depression and dullness, watery diarrhea, and polyurea. The peak of the clinical sign was observed at 7 and 10 dpi. After that, no such signs were observed throughout the experimental period.

### *Gross pathology*

During the 21-day observation, lung and kidney pathology were assessed at 3, 5, 7, 10, 14, 18, and 21 dpi in chickens inoculated with the GI-1 and GI-24 IBV variants. At 3 and 5 dpi, no appreciable gross lesions were observed in either group, and the lungs and kidneys appeared comparable to those of the uninfected controls.

Lung lesions became apparent at 7 dpi and persisted up to 21 dpi (Fig. 3). At 7 dpi, birds in the GI-1 group displayed moderate pulmonary congestion, while those in the GI-24 group showed only mild and focal congestion. By 10 dpi, lungs from the GI-1 infected birds demonstrated pronounced congestion and swelling, and while congestion in the GI-24 group remained comparatively less severe. At 14 dpi, GI-1 lungs showed severe congestion, progressive dark discoloration, focal hemorrhages, and consolidation. GI-24 lungs, however, appeared dark red, less firm, and with a smoother surface. By 18 dpi, prominent consolidation and diffuse congestion persisted in GI-1, whereas GI-24 infected lungs displayed signs of resolution. At 21 dpi, GI-24 infected lungs exhibited residual lesions and mild congestion, while most lung lesions in GI-1 IBV-infected birds had resolved, and the lungs appeared near-normal. Throughout the study, lungs from the control group remained normal, pink, smooth, and free of visible lesions.

**Fig. 3:**
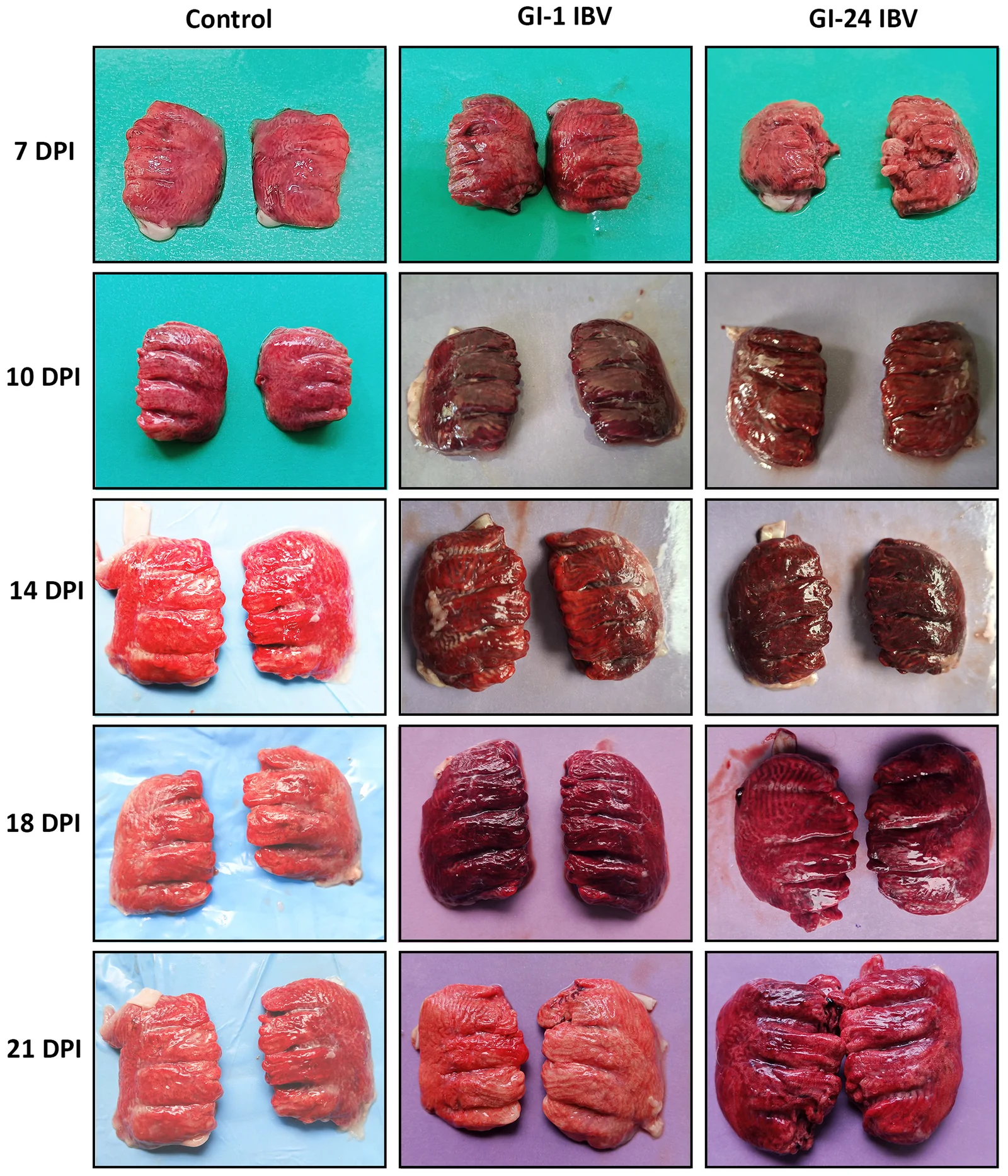
Gross lesions of lungs in broiler chickens from the control, GI-1 IBV- and GI-24 IBV groups were observed at 7, 10, 14, 18, and 21 days post-infection (dpi). Chickens of the GI-1 IBV group showed marked congestion, progressive dark discoloration, and consolidation up to 18 dpi, with partial resolution at 21 dpi. In the GI-24 IBV group, mild to moderate lung congestion was noted at 7 and 10 dpi, progressing to darkening and slight firmness at 14 dpi, followed by gradual resolution of lesions between 18 and 21 dpi. The lungs of the control group maintained normal morphology throughout the experiment.

After experimental infection, both GI-1 and GI-24 IBV groups exhibited gross renal lesions with distinct severity and progression up to 21 dpi (Fig. 4). At 7 dpi, kidneys from the GI-1 IBV group demonstrated mild swelling and focal congestion, with scattered reddish areas indicative of early nephritis. In contrast, the GI-24 IBV group presented with kidneys of nearly normal appearance, displaying only mild hyperemia. By 10 dpi, GI-1 IBV group kidneys showed pronounced enlargement, extensive congestion, and generalized dark discoloration, indicating progressive. Lobulation became obscured due to edema and tissue fragility. GI-24 IBV group kidneys displayed mild to moderate swelling and congestion, but to a lesser extent than GI-1 IBV. At 14 dpi, both GI-1 and GI-24 IBV showed almost similar lesions, like 10 dpi. Kidneys of the GI-1 IBV group remained enlarged with diffuse dark congestion and multiple hemorrhagic spots, while GI-24 IBV group kidneys were moderately swollen, with improved distinction of lobules and reduced congestion at 18 dpi compared to 14 dpi. At 21 dpi, GI-1 IBV kidneys still exhibited severe congestion, patchy hemorrhages, and surface irregularity, suggesting progression to chronic lesions. In contrast, GI-24 IBV group kidneys had regained near-normal size and color, with only rare foci of mild congestion. The control group exhibited consistently normal renal morphology.

**Fig. 4:**
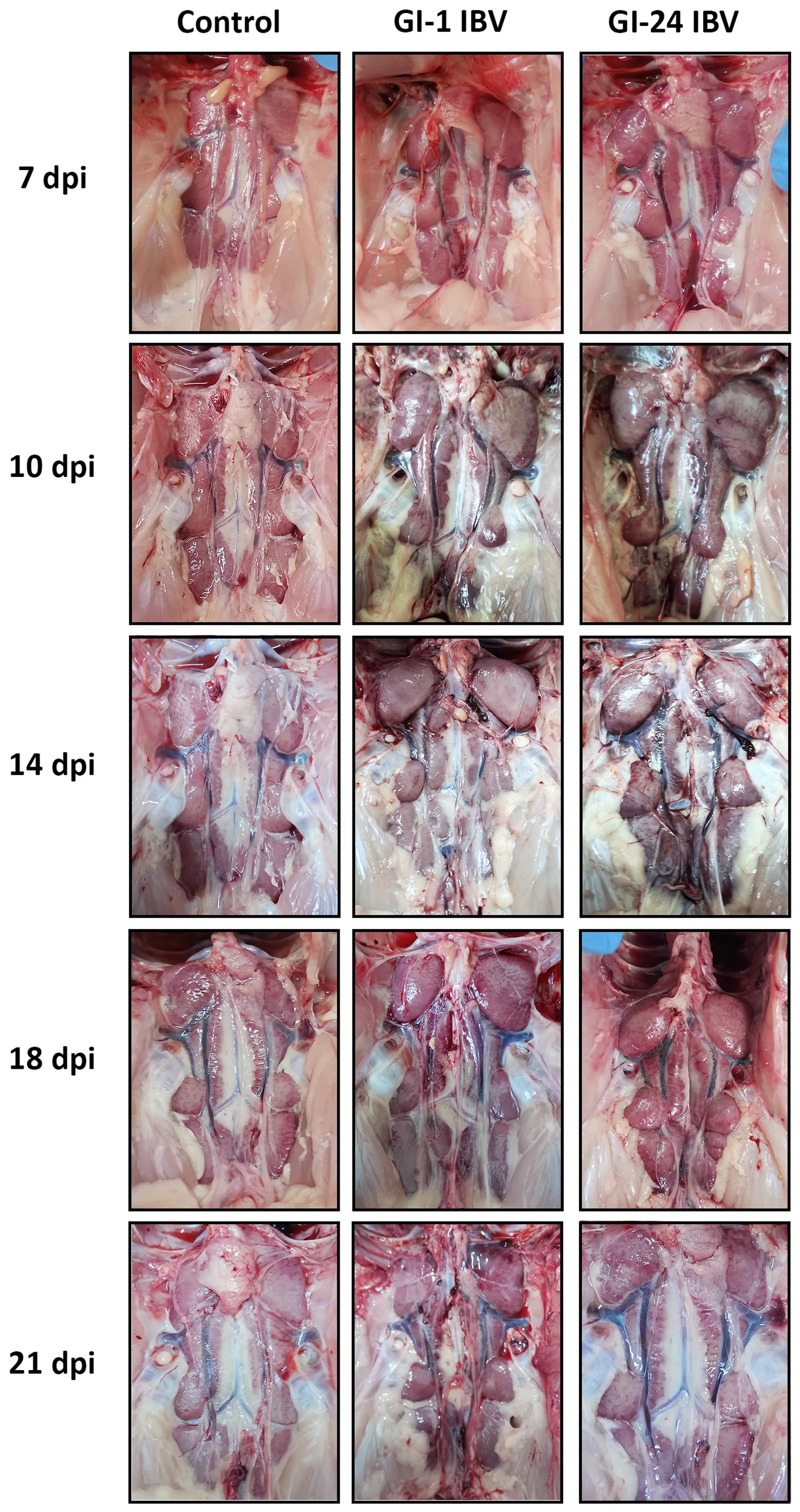
Gross renal lesions in broiler chickens from the control, GI-1 IBV and GI-24 IBV groups were observed at 7, 10, 14, 18, and 21 days post-infection (dpi). Chickens in the GI-1 IBV group exhibited marked renal enlargement, congestion, multifocal hemorrhages, and progressive dark discoloration from 7 to 21 dpi. In the GI-24 IBV group, kidneys showed mild to moderate swelling and congestion, most pronounced at 10–14 dpi, with resolution noted at 21 dpi. Examined kidneys of the control group maintained normal morphology throughout the experiment.

### *Histopathology*

During the 21-day observation, lung and kidney lesions were examined at 3, 5, 7, 10, 14, 18, and 21 dpi in chickens infected with GI-1 and GI-24 IBV. At 3 and 5 dpi, no notable microscopic lesions were detected in either group, and the organs resembled those of the controls.

At 7 dpi, the lungs of the GI-1 IBV group exhibited marked perivascular edema and marked pulmonary congestion, indicating acute inflammatory injury and increased vascular permeability. In contrast, the GI-24 IBV group showed pulmonary edema with only mild congestion, indicating a slightly less severe acute response at this stage. By 10 and 14 dpi, both infected groups demonstrated similar congestion and perivascular edema; however, perivascular edema was mildly more prominent at 14 dpi in the GI-24 IBV group. This pattern reflects a transition from the acute phase to a subacute stage, where vascular changes persist but the intensity of inflammation is reduced. At 18 dpi, the GI-1 IBV group developed features of chronic bronchitis, characterized by persistent inflammation and structural changes in the bronchial walls. The GI-24 IBV group, meanwhile, showed congestion of bronchi and blood vessels, reflecting ongoing but less advanced chronic changes. By 21 dpi, both groups exhibited chronic bronchitis marked by thickening of bronchial walls and chronic inflammatory cell infiltration, consistent with the chronic sequelae of IBV infection. The control group exhibited normal lung histology, with no evidence of congestion, edema, or inflammatory changes (Fig. 5).

**Fig. 5:**
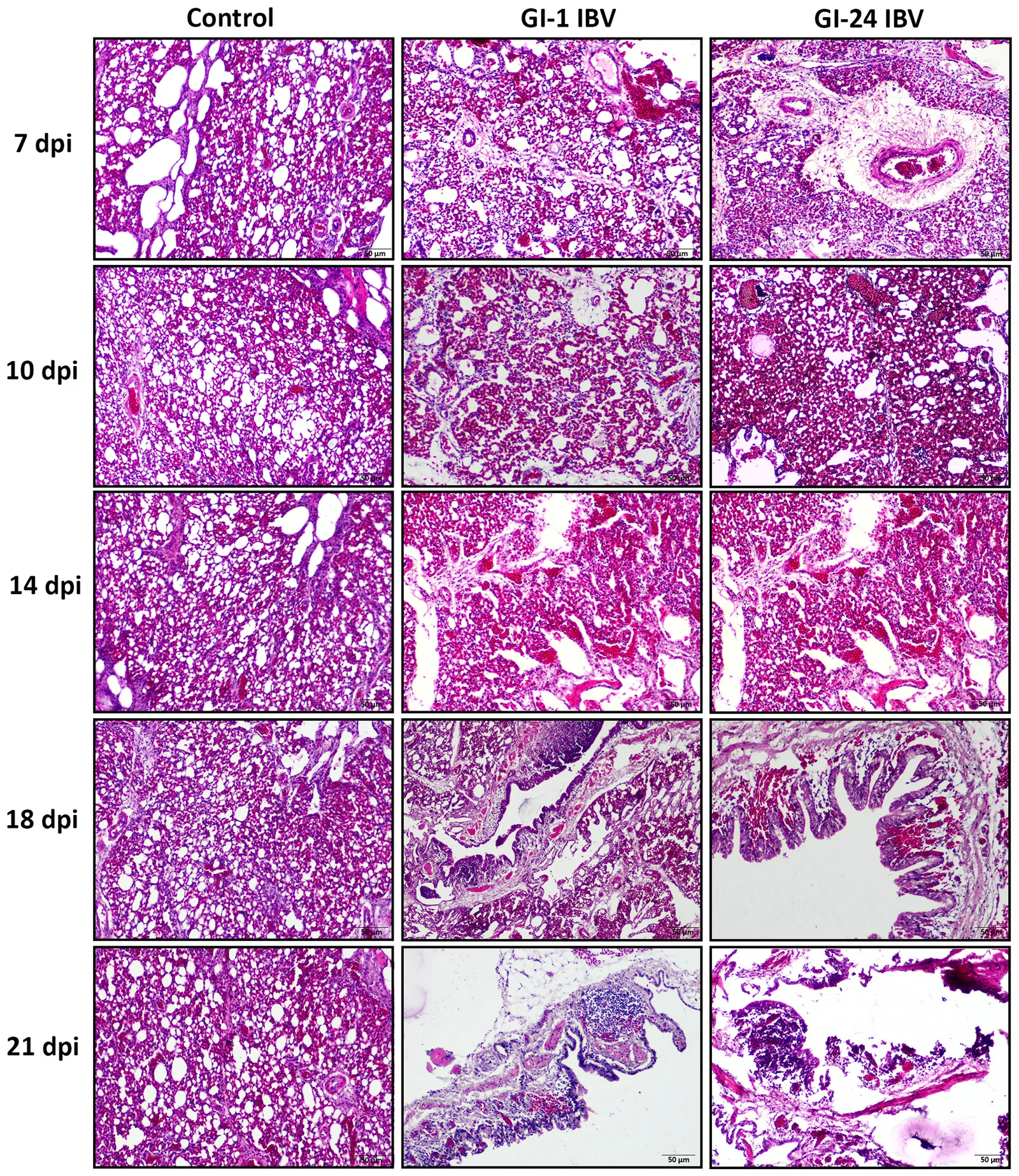
Microscopic lesions of the lungs in broiler chickens from the control, GI-1 IBV and GI-24 IBV groups are shown at 7, 10, 14, 18, and 21 days post-infection (dpi). At 7 dpi, chickens in the GI-1 IBV group showed marked pulmonary congestion and peri‑vascular edema, whereas those in the GI-24 IBV group exhibited mild congestion and peri‑vascular edema. At 10 and 14 dpi, both infected groups exhibited similar slight pulmonary congestion and peri‑vascular edema; however, perivascular edema was mildly more prominent at 14 dpi in the GI-24 IBV group. By 18 dpi, chickens in the GI-1 IBV group displayed histological evidence of chronic bronchitis, while those in the GI-24 IBV group had only congested bronchi. At 21 dpi, both IBV-infected groups exhibited features of chronic bronchitis. Throughout all time points, the control group maintained a normal pulmonary histological architecture. Scale bar indicates the magnification of histopathological slides.

At 7 dpi, kidneys of both GI-1 and GI-24 IBV-infected groups exhibited necrotizing tubular nephritis, characterized by extensive necrosis and inflammation of the renal tubules, indicating acute and severe viral-induced damage. By 10 dpi, the GI-1 IBV group continued to display degenerative tubule-nephritis, marked by ongoing tubular cell degeneration and inflammation, whereas the GI-24 IBV group demonstrated even more pronounced renal hemorrhage, suggesting persistent vascular injury and tissue breakdown. At 14 dpi, the GI-1 IBV group showed evidence of regeneration alongside areas of necrosis and degeneration, indicating the initiation of tissue repair processes while some acute damage persisted. The GI-24 IBV group at this stage exhibited both tubular degeneration and regeneration, reflecting a mixed pattern of ongoing injury and recovery. By 18 dpi, both groups presented with degenerative changes, suggesting substantial resolution of the acute phase, although the necrotizing lesions remained evident. At 21 dpi, both IBV-infected groups showed marked regenerative alterations. The control group maintained normal renal histology throughout the observation period (Fig. 6).

**Fig. 6:**
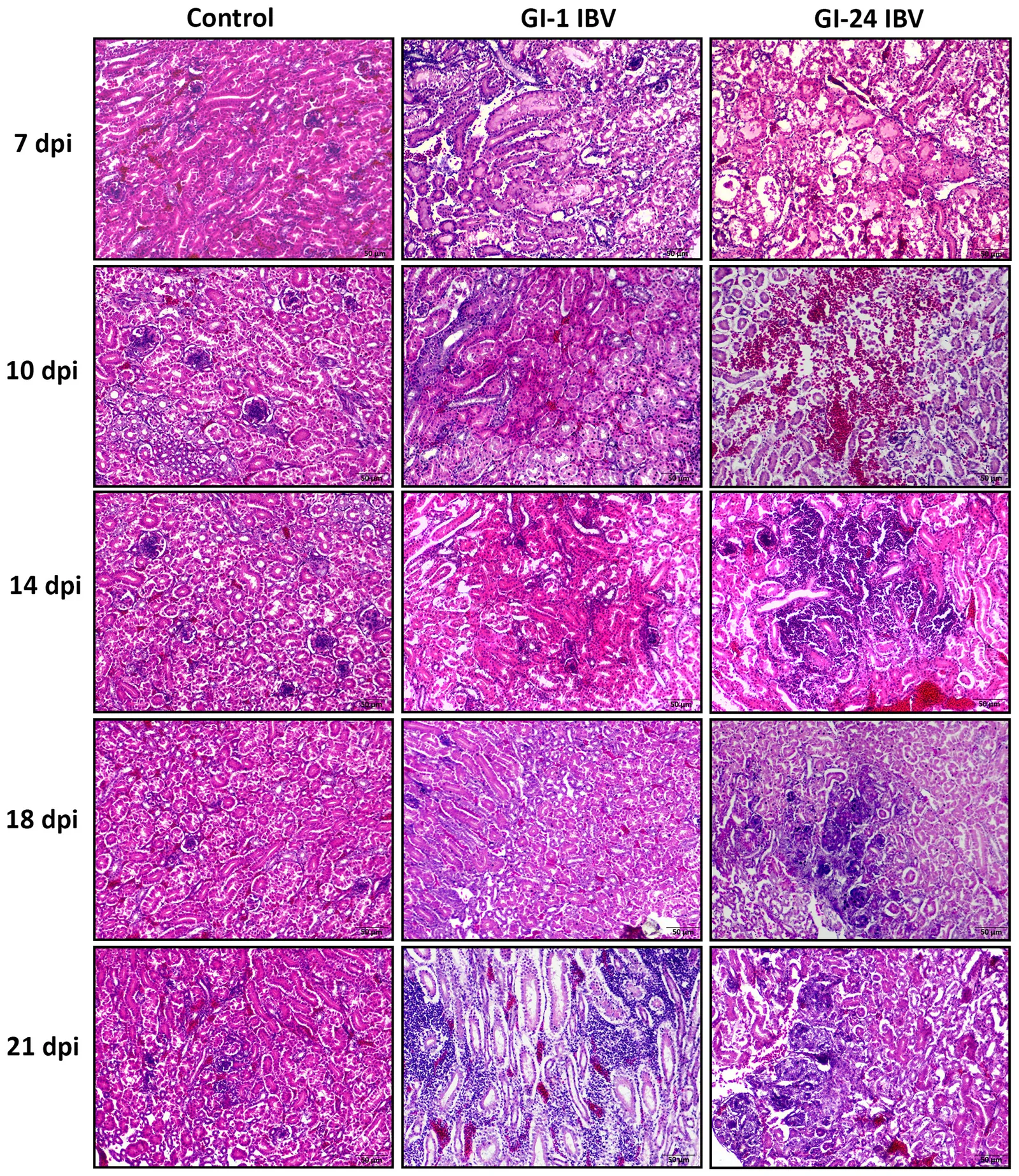
Microscopic lesions of the kidneys were assessed in broiler chickens from the control, GI-1 IBV and GI-24 IBV groups are shown at 7, 10, 14, 18, and 21 days post-infection (dpi). At 7 dpi, both groups exhibited focal necrotizing tubular nephritis. At 10–14 dpi, necrotizing tubule-nephritis persisted in both infected groups, with renal hemorrhages more pronounced in chickens of the GI-24 IBV group. At 18 dpi, regenerative changes emerged in both groups, although necrotizing lesions remained evident. By 21 dpi, kidneys from both infected groups showed marked regenerative alterations, suggesting progressive repair. The control birds exhibited histologically normal renal architecture at all points of the experiment. Scale bar indicates the magnification of histopathological slides.

### *Viral load in swabs and tissues*

Viral RNA was detected in the oropharyngeal and cloacal swabs, respiratory and renal tissues of broiler chickens experimentally infected with two different variants of infectious bronchitis virus (IBV), namely GⅠ−1 and GⅠ−24, during a 21-day post-infection (dpi) period.

In the oropharyngeal swab (Fig. 7A), the viral load profile demonstrated that both GI-1 and GI-24 IBV-infected groups initiated viral shedding at 5 dpi, while all samples from the control group remained negative throughout the study period. Viral shedding increased sharply and reached its peak between 10 and 14 dpi in both infected groups. Notably, the GI-24 variant showed significantly higher viral RNA loads than the GI-1 variant at 5 and 7dpi (*P* < 0.001), indicating an early replication capacity in the upper respiratory tract. Following 14 dpi, viral loads progressively declined in both groups; however, the GI-1 group exhibited slightly prolonged viral persistence, with detectable RNA levels extending up to 21 dpi.

**Fig. 7:**
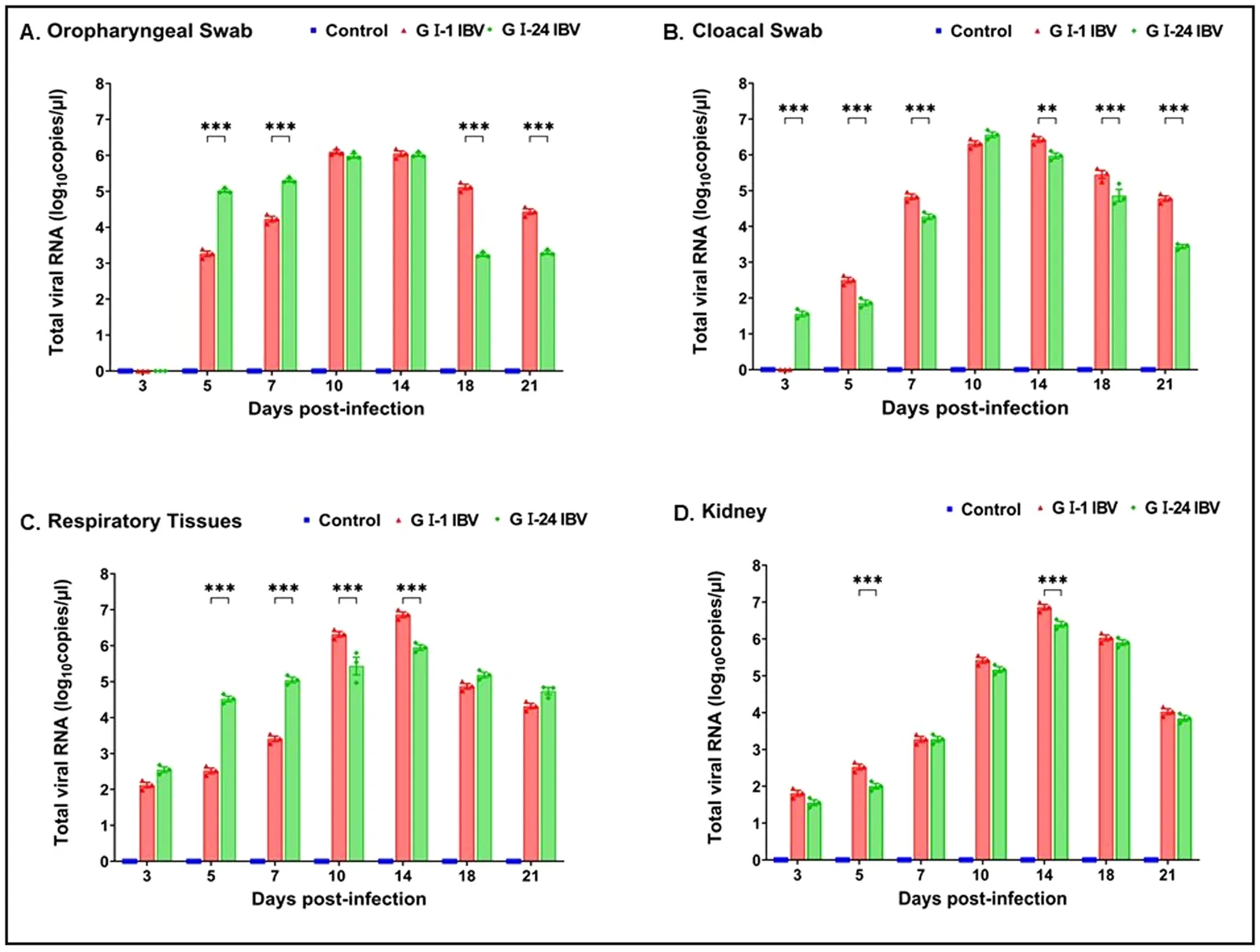
Viral RNA load in swabs and tissues of broiler chickens infected with GI-1 and GI-24 IBV variants compared with the control group; **(A)** oropharyngeal swabs, **(B)** cloacal swabs, **(C)** respiratory tissues, and **(D)** renal tissues. Viral RNA loads were expressed as log₁₀ copies/µl, and values are presented as mean ± SEM. Statistical analysis was performed using Two-way ANOVA with Tukey’s multiple comparison test. Error bars represent mean ± SEM. Asterisks indicate levels of significance as *** *P* ≤ 0.001; ** *P* ≤ 0.002.

Viral load analysis from cloacal swabs (Fig. 7B) showed that viral RNA was first detected in both the GI-1 and GI-24 infected groups at 3 dpi, whereas the control group remained consistently negative throughout the study. Cloacal viral shedding gradually increased and reached its maximum levels between 10 and 14 dpi, indicating systemic dissemination of the virus to the urinary tract. In contrast to the oropharyngeal shedding pattern, the GI-1 variant exhibited consistently higher cloacal viral loads than GI-24 across the observation period, with statistically significant differences (*P* < 0.001), suggesting stronger replication or prolonged persistence in the urinary system. After 14 dpi, viral loads remained detectable in both infected groups up to 21 dpi, with GI-1 maintaining slightly higher levels of shedding than GI-24**.**

In respiratory tissues, viral RNA was first detected at 3 dpi in both the GI-1 and GI-24 infected groups, while all control birds remained negative throughout the study (Fig. 7C). Viral load increased steadily thereafter and reached maximum levels between 14 and 18 dpi, coinciding with the peak phase of respiratory infection. Across early sampling points, the GI-24 variant exhibited significantly higher viral RNA concentrations than GI-1 (*P* < 0.001), demonstrating its early replication kinetics in respiratory tissues. Following 18 dpi, viral loads declined in both groups but remained detectable up to 21 dpi, indicating persistent, though waning, viral replication. Collectively, these findings indicate that IBV variants efficiently replicate in the respiratory tissues.

In renal tissues, viral RNA was detectable from 3 dpi onward in both GI-1 and GI-24 infected groups compared to the control group (Fig. 7D). Viral loads increased progressively and peaked at 14 dpi in both variants, after which they declined but remained detectable up to 21 dpi. Although the GI-1 variant exhibited significantly higher renal viral RNA levels than GI-24 (5 and 14 dpi; *P* ***<*** ***0.001***), no significant differences were observed at the remaining time points. These data indicate that both variants appear to possess similar renal pathogenic potential.

### *Immune response after infection*

The serological immune response against infectious bronchitis virus (IBV) was evaluated in the GI-1 and GI-24 IBV groups and the control group (Fig. 8). At 7 days post-infection (dpi), all groups exhibited low ELISA titers, with values remaining below the assay cut-off. By 14 dpi, both the GI-1 and GI-24 IBV groups showed markedly elevated ELISA titers that exceeded the cut-off value, indicating a strong antibody-mediated immune response. At 21 dpi, ELISA titers in both IBV-infected groups increased further, with the GI-1 IBV group showing the highest titers, followed by the GI-24 IBV group; both were significantly higher than those of the control group.

**Fig. 8:**
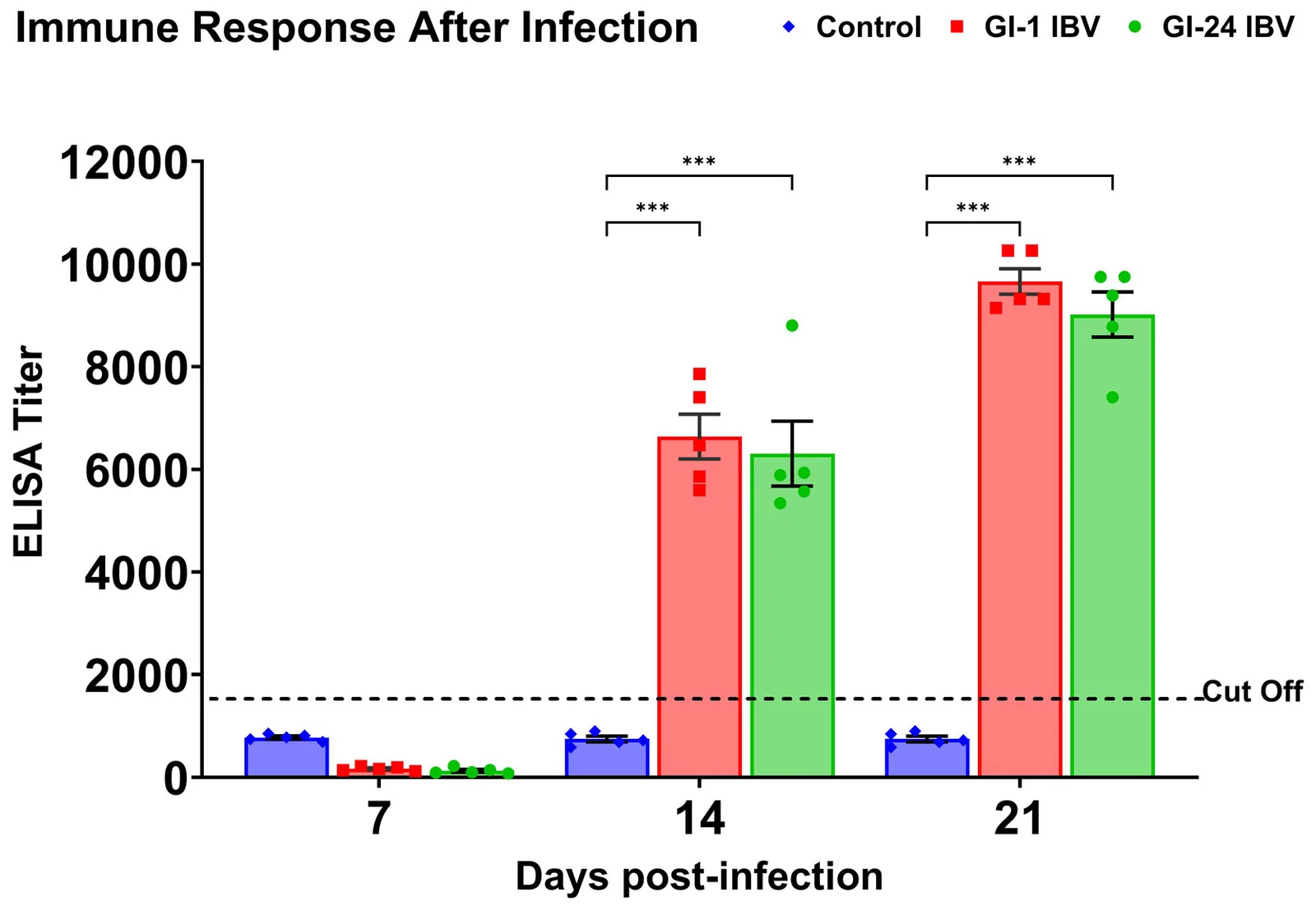
Immune responses after infection as measured by ELISA titer in control, GI-1 IBV, and GI-24 IBV groups. Bars represented mean ELISA titers, standard error of the mean (SEM). Asterisks indicated significant differences (∗∗∗=*P* < 0.001).

### *Dynamics of cytokines expression in respiratory tissues following IBV infection*

The expression dynamics of pro‑inflammatory cytokines (IL‑1β and TNF‑α), antiviral interferons (IFN‑β and IFN‑γ), and the anti‑inflammatory cytokine IL‑10 in respiratory tissues are illustrated in Fig. 9. IL‑1β expression increased significantly in both GI‑1‑ and GI‑24‑infected broiler chickens, peaking at 14 days post‑infection (dpi) compared with uninfected controls. Notably, broilers infected with the GI‑1 strain exhibited significantly higher (*P* < 0.001) IL‑1β expression levels at 7, 10, and 14 dpi than those infected with the GI‑24 variant (Fig. 9A). A significant upregulation (*P* < 0.01) of TNF‑α expression was observed at 10, 14, and 21 dpi in broiler chickens infected with the GI‑1 strain compared with the GI‑24 variant, with maximal expression recorded at 14 dpi. Thereafter, TNF‑α levels declined, reaching minimal expression by 21 dpi (Fig. 9B).

**Fig. 9:**
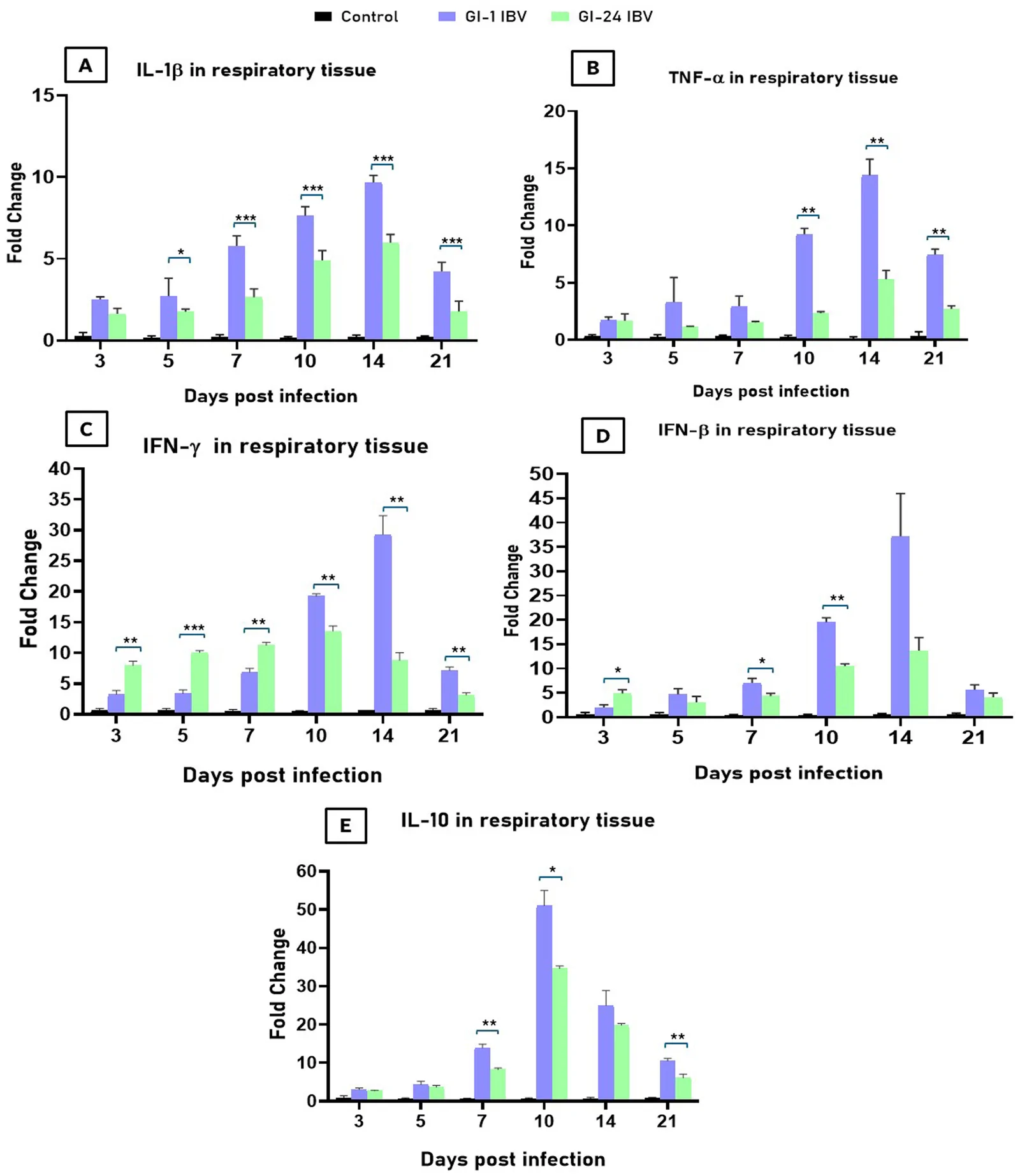
Expression of proinflammatory (IL-1β and TNF-α) and antiviral (IFN-β, IFN-γ and IL-10) cytokines in the respiratory tissues (pooled trachea and lungs) (**A-E**) of broiler chickens of control, GI-1 and GI-24 IBV infected groups. Expression levels were normalized to the housekeeping gene GAPDH, which was validated for stable expression across groups (Control and infected groups). Bar diagram indicates the relative expression of IL-1β (A), TNF-α (B), IFN-β (C), IFN-γ (D) and IL-10 (E) cytokines. Statistical analysis was performed with Two-way ANOVA with Tukey’s multiple comparison test where X-axis denotes days post infection and Y-axis denotes fold changes. Error bars represent the mean ± SD. Star indicates level of significance as ***P ≤ 0.001, **P ≤ 0.01, * 0.01 < *P* < 0.05.

In respiratory tissues, the antiviral cytokine IFN‑γ was significantly upregulated (*P* < 0.01) from 3 dpi and remained elevated through 21 dpi in both IBV‑infected groups. During the early phase of infection (up to 7 dpi), broiler chickens infected with the GI‑24 variant exhibited higher IFN‑γ expression than those infected with the GI‑1 strain. This elevated expression gradually declined toward 21 dpi. In contrast, GI‑1 infection induced a more pronounced IFN‑γ response at 14 dpi, followed by a gradual reduction by 21 dpi (Fig. 9C). The expression of IFN‑β remained relatively stable at 3, 5, and 7 dpi in both infection groups but showed a significant peak at 14 dpi, indicating a delayed yet robust antiviral response in respiratory tissues (Fig. 9D).

The anti‑inflammatory cytokine IL‑10 was significantly upregulated (*P* < 0.01) at 10 dpi in the respiratory tissues of GI‑1‑infected broiler chickens, followed by a gradual decline through 21 dpi. A similar expression pattern was observed in birds infected with the GI‑24 variant, suggesting comparable regulatory immune responses induced by both strains (Fig. 9E).

### *Dynamics of cytokines expression in kidney following IBV infection*

Following experimental infection, both GI‑1 and GI‑24 IBV variants induced robust but distinct cytokine expression profiles in renal tissues (Fig. 10). The pro‑inflammatory cytokine IL‑1β showed sustained upregulation in both infected groups, with peak expression observed at 10 and 14 days post‑infection (dpi). Notably, the GI‑1 variant induced a significantly higher magnitude of IL‑1β expression compared with the GI‑24 variant (*P* < 0.001; Fig. 10A). At 3, 5, 7, and 21 dpi, IL‑1β expression in all infected groups was significantly elevated (*P* < 0.05) relative to uninfected controls, with marked differences between IBV variants at 3, 7, and 21 dpi (*P* < 0.001).

**Fig. 10:**
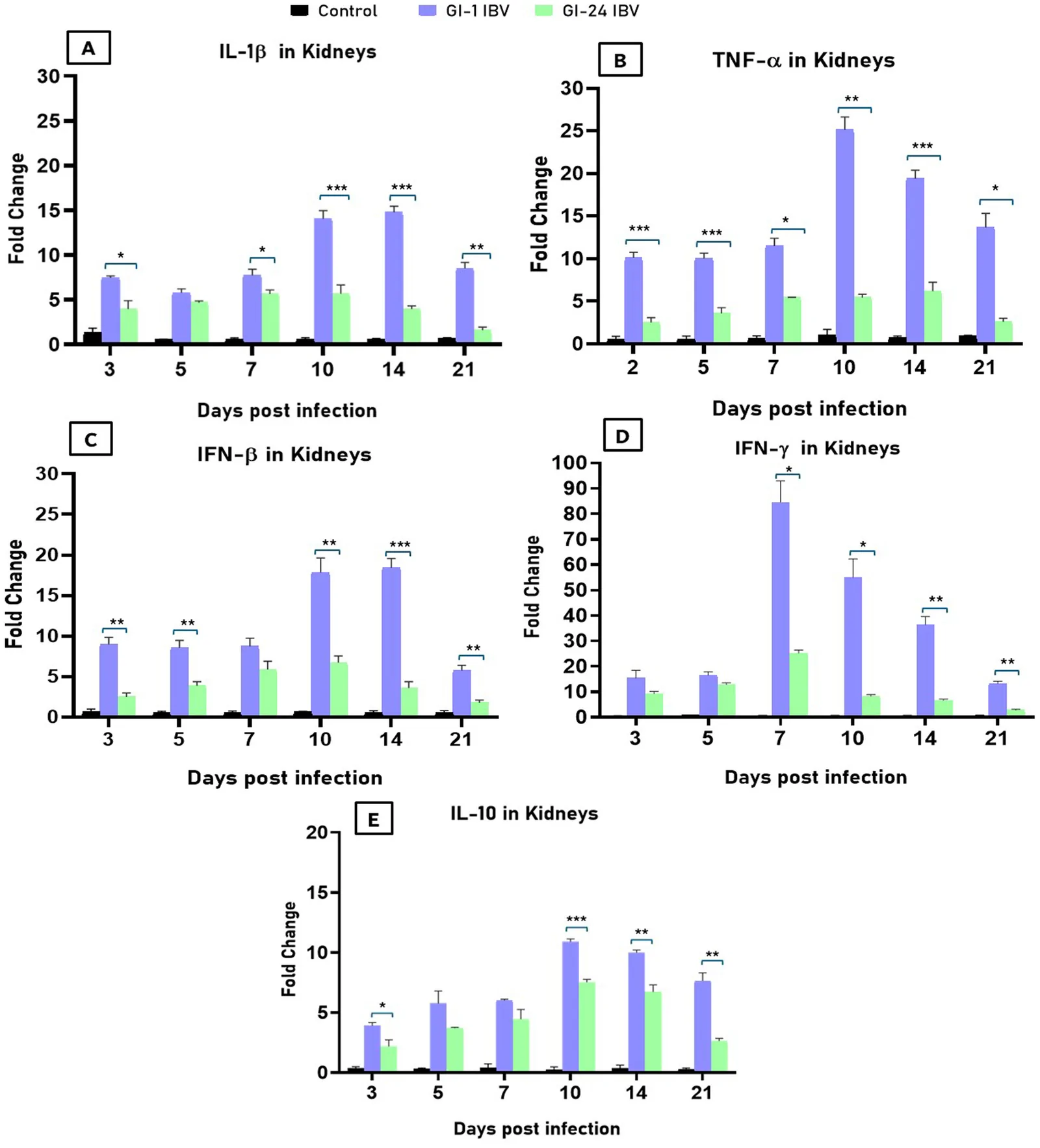
Expression of proinflammatory (IL-1β and TNF-α) and antiviral (IFN-β, IFN-γ and IL-10) cytokines in the renal tissues (**A-E**) of broiler chickens of control group, GI-1 and GI-24 IBV infected groups. Expression levels were normalized to the housekeeping gene GAPDH, which was validated for stable expression across groups (Control and infected groups). Bar diagram indicates the relative expression of IL-1β (A), TNF-α (B), IFN-β (C), IFN-γ (D) and IL-10 (E) cytokines. Statistical analysis was performed with Two-way ANOVA with Tukey’s multiple comparison test where X-axis denotes days post infection and Y-axis denotes fold changes. Error bars represent the mean ± SD. Star indicates level of significance as ***P ≤ 0.001, **P ≤ 0.01, * 0.01 < *P* < 0.05.

The expression of TNF‑α in kidneys was significantly upregulated in all infected groups compared with controls, reaching peak levels at 10 dpi (*P* < 0.001; Fig. 10B). Across all examined time points, broiler chickens infected with the GI‑1 variant consistently exhibited higher TNF‑α expression than those infected with the GI‑24 variant (*P* < 0.01), indicating a stronger pro‑inflammatory response associated with GI‑1 infection.

Renal expression of the antiviral cytokine IFN‑γ was significantly increased from 3 dpi onward in both IBV‑infected groups (*P* < 0.01); however, peak expression differed between variants. In GI‑24‑infected birds, IFN‑γ expression reached its maximum at 10 dpi, whereas GI‑1‑infected broilers exhibited a delayed peak at 14 dpi. IFN‑γ levels gradually declined in both groups by 21 dpi (Fig. 10C). In contrast, IFN‑β expression remained relatively stable during the early phase of infection (3–5 dpi) but showed a significant increase at 7 dpi in both infected groups (*P* < 0.01). This response was more pronounced in broiler chickens infected with the GI‑1 variant compared with the GI‑24 variant (Fig. 10D).

The anti‑inflammatory cytokine IL‑10 was significantly upregulated in the kidneys of GI‑1‑infected broiler chickens at 3, 10, 14, and 21 dpi compared with GI‑24‑infected birds (*P* < 0.01; Fig. 10E), suggesting a stronger regulatory immune response associated with GI‑1 infection. Overall, IBV infection triggered strong, time‑dependent pro‑inflammatory and antiviral cytokine responses in both respiratory and renal tissues, with the GI‑1 variant consistently inducing more intense and sustained immune activation than the GI‑24 variant. The pronounced and prolonged cytokine upregulation in kidneys, particularly for IL‑1β, TNF‑α, IFN‑γ, and IL‑10, underscores the marked nephrotropic nature of IBV and highlights variant‑specific immune modulation contributing to renal pathology.

## Discussion

The poultry industry in Bangladesh plays a vital role in the national economy and food security, producing millions of broiler and layer chickens annually (Uddin et al., 2025). However, infectious bronchitis virus (IBV) continues to cause frequent outbreaks in both commercial and backyard poultry, resulting in substantial economic losses and impaired growth performance (Parvin et al., 2021). Among the circulating genotypes, GI-1 (Mass-type) is one of the most widely distributed and historically well-characterized IBV lineages worldwide. This classical Mass-type variant predominantly targets the respiratory system, leading to significant respiratory disease in chickens (Cavanagh, 2007).

In recent years, the emergence of novel IBV variants has complicated disease control. The GI-24 variant has been reported recently in neighboring countries, including India in 2024 and Pakistan in 2023 (Rafique et al., 2024; Saleem et al., 2024). Recent field investigations in Bangladesh have identified the dominant circulation of GI-1 lineages alongside the first report of the GI-24 genotype (Kabir et al., 2026).

The present study provides a comprehensive comparison of the pathogenesis, tissue tropism, viral persistence, and immune responses associated with GI-1 and GI-24 IBV variants in broiler chickens. Previous S1 sequence analysis identified genetic and predicted antigenic differences between GI-24 and commonly used vaccine strains, particularly within S1 hypervariable and predicted neutralizing epitope regions (Kabir et al., 2026) . These findings suggest potential antigenic divergence that may contribute to reduced heterologous protection (Bhuiyan et al., 2019; Uddin et al., 2025). The identification of GI-24 as a newly circulating variant in Bangladesh further underscores the continuous evolutionary diversification of IBV and highlights the vulnerability of current vaccination programs to emerging genotypes. Understanding the biological behavior of these variants is essential for designing effective vaccination and control strategies adapted to the country’s specific epidemiological conditions.

Clinical evaluation demonstrated that both GI-1 and GI-24 infections induced a clear febrile response beginning at 3 dpi, peaking at 7-10 dpi, and resolving by 14-18 dpi, consistent with the acute phase of IBV infection. The slightly higher and more prolonged fever observed in the GI-1 group suggests stronger or more sustained inflammatory activation. Similar febrile patterns have been reported during early IBV infection and are commonly associated with peak viral replication and cytokine induction (Yan et al., 2021; Alhafufi et al., 2025). The absence of temperature fluctuations in the control group confirms that the observed pyrexia was infection-specific.

Both GI-1 and GI-24 infections resulted in significant and sustained reductions in body-weight gain compared with controls, beginning from 5 dpi and persisting through 21 dpi. Although GI-24-infected birds exhibited a slightly greater early reduction, both infected groups failed to achieve normal growth trajectories, reflecting the lasting production impact of IBV. These findings align with previous reports demonstrating that IBV-associated respiratory compromise and renal dysfunction impair feed intake and nutrient utilization (Yan et al., 2016; Manswr et al., 2020). The persistent growth deficit underscores the economic consequences of IBV even in the absence of mortality.

Clinically, infected birds exhibited typical IBV‑associated respiratory signs such as coughing, sneezing, nasal discharge, and tracheal rales alongside depression and renal manifestations. Clinical signs appeared as early as 3 dpi and persisted up to 10 dpi, whereas control birds remained clinically normal. These observations are consistent with established descriptions of IBV infection, which frequently involves simultaneous respiratory and renal involvement, particularly with nephropathogenic variants (Yan et al., 2016; Ishag et al., 2024; Sadek et al., 2024). The absence of mortality suggests moderate virulence under experimental conditions but does not diminish the clinical and production significance of the infections.

Gross and histopathological evaluation of lung tissues revealed variant-dependent differences in lesion severity and duration. GI-1 infection induced more pronounced pulmonary congestion, consolidation, and chronic bronchitis that persisted up to 21 dpi. Additionally, GI-24-infected birds also displayed significant respiratory lesions alongside nephrotropism that resolved earlier. Detailed pulmonary pathology associated with GI-24 has been minimally reported (Mahana et al., 2019; Saleem et al., 2024; Sharma et al., 2024). However, the present findings suggest that this GI-24 strain exhibits stronger respiratory replication.

Renal pathology further distinguished the two variants. GI-1 infection resulted in severe and persistent nephropathology, characterized by kidney enlargement, congestion, hemorrhage, tubular necrosis, and delayed regeneration. Conversely, GI-24 induced milder renal lesions that peaked between 10 and 14 dpi and largely resolved by 21 dpi. Histologically, acute necrotizing tubular nephritis with inflammatory infiltration predominated in GI-1-infected kidneys, whereas GI-24 infection showed mixed degenerative and regenerative changes. These lesions are characteristic of nephropathogenic IBV and align with previous experimental and field observations (Mahana et al., 2019; Saleem et al., 2024; Sharma et al., 2024).

Viral-load analysis demonstrated that both GI-1 and GI-24 replicated efficiently in respiratory and renal tissues and were shed via oropharyngeal and cloacal routes from as early as 3 dpi.

Although GI-24 showed higher viral loads in respiratory tissues and oropharyngeal swabs during peak infection, renal viral kinetics were largely comparable between variants, with persistence up to 21 dpi. Thus, the GI‑24 isolate used in our study induced significant renal pathology, as well as it also demonstrated pronounced respiratory tropism, as evidenced by respiratory lesions, viral load distribution, and prolonged respiratory shedding. This suggests that the Bangladeshi GI‑24 isolate possesses broader tissue tropism than previously recognized. Previous pathogenicity studies in Asia have primarily focused on IBV genotypes such as GI-1, GI-13, GI-19, GI-23 and GI-29 (Jiang et al., 2017; Yan et al., 2021 and Yan et al., 2023). In contrast, the present study investigated the recently emerged GI-24 genotype, commonly referred to as the Indian variant (Raja et al., 2020), for which information on pathogenesis and host immune responses remains limited. Although GI-24 has previously been associated mainly with nephropathogenicity, our findings demonstrate that this genotype exhibits both respiratory and renal tropism. Furthermore, to our knowledge, this is the first study to characterize inflammatory and pro-inflammatory cytokine responses induced by a GI-24 strain in specific target organs as well as oropharyngeal and cloacal swab samples. These findings provide novel insights into the pathogenic and immunomodulatory properties of this emerging genotype.

A possible explanation for the differential tissue tropism is variation in receptor availability between respiratory and renal tissues. IBV interacts with host-cell sialic acid receptors through the S1 protein, and differences in receptor distribution may influence tissue-specific viral replication. The higher early respiratory viral load of GI-24 may therefore reflect more efficient replication in respiratory epithelium, whereas the later renal persistence observed for GI-1 may indicate greater adaptation to renal tissues. Differences in S1 sequences between GI-1 and GI-24, including substitutions in the receptor-binding region, may also contribute to these differences (Kabir et al., 2026). However, receptor expression and binding were not directly assessed in this study; thus, this proposed mechanism remains speculative and requires further investigation. Overall, despite minor differences in tissue preference and lesion dynamics, GI-1 and GI-24 demonstrated comparable pathogenicity under experimental conditions.

This study also discussed the immune response against IBV after infection. The serological data demonstrates a classic antibody-mediated immune response following IBV infection. Both GI-1 and GI-24 IBV-infected groups showed low ELISA titers at 7 dpi, with a marked increase by 14 dpi and further elevation at 21 dpi, while the control group remained seronegative throughout. The GI-1 IBV group exhibited the highest antibody titers at 21 dpi, though the difference with GI-24 was not statistically significant. This pattern is consistent with established IBV immunology, where specific antibody responses typically become detectable by ELISA around 10–14 days post-infection, peaking in the third week, which is a classic feature of humoral immunity in response to IBV infection (Manswr et al., 2020). However, the lack of significant differences between GI-1 IBV and GI-24 IBV suggests both variants are immunogenic, and host factors or viral genetics may modulate the magnitude of response.

Although GI-1 is generally regarded as a recombinant vaccine-derived variant typically associated with subclinical infection, it induced more progressive clinical signs in this study, accompanied by persistent respiratory and renal lesions. This enhanced pathogenicity was associated with altered tissue tropism, particularly increased renal involvement, suggesting that genetic recombination may have modified its biological behavior beyond that of classical vaccine-related strains. In contrast, GI-24 traditionally described as a nephrotropism strain exhibited relatively stronger respiratory tropism. However, both GI-1 and GI-24 demonstrated comparable viral persistence in the lungs and kidneys, confirming their pronounced pneumotropic and nephrotropic potential and emphasizing the multifaceted tissue affinity of contemporary IBV variants.

The immunopathogenesis of IBV is characterized by a delicate balance between interferon-mediated viral clearance and pro-inflammatory-mediated tissue damage (Newton et al., 2016). The observed pathological differences were closely mirrored by distinct cytokine expression profiles in respiratory and renal tissues. The respiratory tissues of broiler chickens of both infected groups exhibited a synchronized peak of IL-1β, TNF-α, and IFN-β at 14 dpi, are potent drivers of vascular permeability and the recruitment of inflammatory cells (Kameka et al., 2014; Okino et al., 2017; Shila et al., 2026). This early surge of IFN-γ along with significant upregulation of IL-10 at 10 dpi in GI-24 infected broiler chickens coincided with milder respiratory lesions and earlier resolution, despite higher early viral loads. Conversely, prolonged elevation of IFN-γ with delayed IL-10 peak in GI-1 IBV infected birds facilitated severe lung lesions including congestion and hemorrhages. This complexity contrasts with other respiratory viruses (other types of coronaviruses and avian influenza viruses) that significantly downregulate and/or upregulate (at 3 dpi) the relative expression of IFN-γ, and IL-1β during the initial phase of IBV infection within the respiratory tissues (Kameka et al., 2014; Okino et al., 2017; Shila et al., 2026)

GI-1 infection consistently triggered stronger and more sustained pro-inflammatory (IL-1β, TNF-α) and antiviral (IFN-γ, IFN-β) responses than GI-24, particularly in renal tissues. This heightened cytokine activity correlated with increased lesion severity and delayed tissue regeneration, supporting the concept that excessive or prolonged immune activation contributes to IBV-induced immunopathology. Conversely, the relatively milder and transient lesions associated with GI-24 infection aligned with a more regulated cytokine response, despite its effective viral replication. The significant upregulation of IL-10, especially in GI-1-infected kidneys, further suggests an attempt by the host to counterbalance inflammation, although this regulatory response appeared insufficient to prevent tissue damage.

Collectively, these findings highlight the critical role of cytokine-mediated immune responses in shaping IBV disease outcomes and demonstrate that pathogenicity is not solely determined by viral load or classical tropism classification. Instead, variant-specific immune modulation, coupled with prolonged viral persistence in target organs, appears to drive disease severity and tissue injury. The emergence of GI-24 in Bangladesh, together with the extended pathogenic behavior of GI-1, emphasizes the necessity for continuous molecular surveillance, refined pathogenicity assessment of circulating strains, and the development of strain-matched or broadly protective vaccination strategies to improve IBV control in poultry production systems.

## Conclusion

The present study demonstrates that both GI-1 and GI-24 IBV variants are capable of causing significant respiratory and renal disease in broiler chickens, characterized by extensive tissue damage, prolonged viral shedding, and robust humoral immune responses. However, GI-1 exhibited greater pathogenicity, inducing severe and persistent lesions, higher viral burdens, and stronger inflammatory and antiviral cytokine responses than GI-24. Importantly, although GI-24 has previously been regarded primarily as a nephropathogenic genotype, the Bangladeshi GI-24 isolate displayed dual respiratory and renal tropism, indicating a broader tissue distribution than previously recognized. Furthermore, this study provides the first detailed characterization of organ-specific and swab-associated inflammatory and pro-inflammatory cytokine responses following GI-24 infection, offering new insights into its immunopathogenesis. Collectively, these findings enhance our understanding of the biological behavior of emerging GI-24 variants and highlight the importance of continuous molecular surveillance, pathogenicity assessment, and evaluation of genotype-matched vaccination strategies to improve IBV control in poultry production.

## Ethics statement

The animal experiments with infectious viruses were handled in the enhanced biosafety level 2 (eBSL-2) facilities at Bangladesh Agricultural University, Mymensingh, following the standard biosafety guidelines. The protocol of the animal studies was examined and approved by the ethical committee of the Bangladesh Agricultural University Research System under the approval number BAURES/ESRC /ET_28122.

## Author Contribution

All authors made substantial contributions to the study's design, data acquisition, analysis, or interpretation; drafted or critically revised the manuscript; approved the final version; and agreed to be accountable for all aspects of the work. **Most. Shahana Akter**: Substantive input into the conception and design of the work, contributed to data acquisition, analysis, and interpretation, drafted the manuscript, critically revised it, and approved the final version. **Mohosin Kabir**: Contributed to data acquisition, analysis, and interpretation, drafted the manuscript, critically revised it, and approved the final version. **Md. Riabbel Hossain**: Contributed to data acquisition, analysis, and interpretation, drafted the manuscript, critically revised it, and approved the final version. Shadia Tasnim: Contributed to data acquisition, analysis, and interpretation, drafted the manuscript, critically revised it, and approved the final version. **Farhana Firoze Promi**: Contributed to data acquisition, analysis, and interpretation, drafted the manuscript, critically revised it, and approved the final version. **Rupaida Akter Shila**: Contributed to data acquisition, analysis, and interpretation, drafted the manuscript, critically revised it, and approved the final version. **Jahan Ara Begum**: Contributed to data acquisition, analysis, and interpretation, supervised the project, drafted and critically revised the manuscript, and approved the final version. **Emdadul Haque Chowdhury**: Contributed to data acquisition, analysis, and interpretation, supervised the project, drafted and critically revised the manuscript, and approved the final version. **Rokshana Parvin**: Conceived and designed the study, acquired funding and resources, contributed to data acquisition, analysis, and interpretation, supervised the project, drafted and critically revised the manuscript, and approved the final version. All authors approved the final manuscript and agreed to be accountable for the accuracy and integrity of the work.

## Funding

This research was funded by CEVA (Ceva Sante Animale), France.

## Disclosures

The authors declare that they have no known competing financial interests or personal relationships that could have appeared to influence the work reported in this paper.

## Footnotes

Supplementary material associated with this article can be found, in the online version, at doi:10.1016/j.psj.2026.107782.
