# Supplementary material for: Comparative pathogenesis, tissue tropism, and immune dynamics of infectious bronchitis virus genotypes GI‑1 and GI‑24 in broiler chickens
Source: Poult Sci. 2026 Sep 15;105(12):107782. doi: 10.1016/j.psj.2026.107782 (PMC13625773; doi:10.1016/j.psj.2026.107782)
Supplement: Supplementary file 1 [file mmc1.docx]

**Supplementary Table 1:** List of primers used for detection of cytokines expression in different organs of IB infected broilers in the experimental study

| **Gene** | **Sequence (5`- 3`)** | | **Reference** |
| --- | --- | --- | --- |
| GAPDH | 343F | GACGTGCAGCAGGAACACTA | **Nang *et al.,* 2011** |
|  | 370R | TCTCCATGGTGGTGA AGACA |  |
| IFN-β | 421F | GCTCACCTCAGCATCAACAA |  |
|  | 607R | GGGTGTTGAGACGTTTGGAT |  |
| IFN-γ | 320F | TGAGCCAGATTGTTTCGATG |  |
|  | 471R | CTTGGCCAGGTCCATGATA |  |
| TNF-α | 57F | CTTCTGAGGCATTTGGAAGC |  |
|  | 407R | ACTGGGCGGTCATAGAACAG |  |
| IL-1β | 134F | GGATTCTGAGCACACCACAGT |  |
|  | 405R | TCTGGTTGATGTCGAAGATGTC |  |
| IL-10 | 249F | GCTGCGCTTCTACACAGATG |  |
|  | 451R | TCCCGTTCTCATCCATCTTC |  |
